# Geographic Variation of Intrahepatic Cholangiocarcinoma, Extrahepatic Cholangiocarcinoma, and Hepatocellular Carcinoma in the United States

**DOI:** 10.1371/journal.pone.0120574

**Published:** 2015-04-02

**Authors:** Sean F. Altekruse, Jessica L. Petrick, Alicia I. Rolin, James E. Cuccinelli, Zhaohui Zou, Zaria Tatalovich, Katherine A. McGlynn

**Affiliations:** 1 Division of Cancer Control and Population Sciences, National Cancer Institute, Rockville, Maryland, United States of America; 2 Division of Cancer Epidemiology and Genetics, National Cancer Institute, Rockville, MD, United States of America; 3 Information Management Services, Inc., Calverton, MD, United States of America; Kaohsiung Chang Gung Memorial Hospital, TAIWAN

## Abstract

**Background:**

Intrahepatic (ICC) and extrahepatic cholangiocarcinomas (ECC) are tumors that arise from cholangiocytes in the bile duct, but ICCs are coded as primary liver cancers while ECCs are coded as biliary tract cancers. The etiology of these tumors is not well understood. It has been suggested that the etiology of ICC is more similar to that of another type of liver cancer, hepatocellular carcinoma (HCC), than to the etiology of ECC. If this is true, geographic incidence patterns and trends in ICC incidence should be more similar to that of HCC than ECC.

**Methods:**

To examine this hypothesis, data from the North American Association of Central Cancer Registries Cancer in North America data file were analyzed. Incidence rates and joinpoint trends were calculated by demographic subgroup. County-level incidence rates were mapped.

**Results:**

Overall incidence rates, racial distribution, male:female ratio, and peak ages were more similar between ICC and ECC than with HCC. During 2000–2009, average annual incidence rates of ECC increased. During 2005–2009, average annual ICC incidence rates also increased. High rates for all three cancer sites were found in the Pacific region, particularly Hawaii and Alaska. Rates of ICC and ECC were also high in the Northeast and the upper Midwest, while rates of HCC were high in the South.

**Conclusions:**

Demographic patterns and geographical variation were more closely related between ICC and ECC than HCC, suggesting that the etiology of ICC and ECC may be similar. Increasing rates of both tumors suggest that further etiology studies are warranted.

## Introduction

Cholangiocarcinomas, tumors that arise from the epithelial cells of the bile duct, are classified by the *International Classification of Diseases for Oncology* (ICD-O) [1] as being either intrahepatic (ICC; ICD-O C22.1) or extrahepatic (ECC; ICD-O C24.0), depending on their location in the bile duct. Because ICCs arise in the part of the bile duct that is inside the liver, they are considered by the ICD-O to be liver tumors (ICD-O C22). As such, ICCs are the second most frequently occurring type of liver cancer in the world, after hepatocellular carcinoma (HCC). In contrast, ECCs arise in the part of the bile duct that lies outside the liver and are classified as biliary tract tumors (ICD-O C24) [1].

Risk factors are not well delineated for either type of cholangiocarcinoma. Both tumors are associated with preexisting medical conditions such as Caroli’s disease, primary sclerosing cholangitis, and inflammatory bowel disease. In some developing countries, liver flukes are also a risk factor for both tumors [2]. Despite these similarities, differences in risk factors have also been reported. Accumulating evidence suggests that hepatitis C virus (HCV), a major risk factor for HCC, may also be an important risk factor for ICC [3–7] but not for ECC [6,7]. Other HCC risk factors, such as obesity, chronic non-alcoholic liver disease, and tobacco, have also been shown to be associated with ICC but not ECC [6]. Geographic variation in the distribution of these risk factors could affect incidence patterns of liver cancer types in the United States (U.S.).

Further indications of differences in ICC and ECC in the U.S. are that incidence rates of ICC, similar to those of HCC, have been increasing even when accounting for misclassification of hilar cholangiocarcinoma (i.e., Klatskin tumors), while incidence rates of ECC have remained fairly stable [8]. If the risk factors for ICC are more similar to the risk factors for HCC than ECC, the geographical incidence patterns for ICC should also be more similar to those of HCC than ECC. To test this hypothesis, incidence rates for ICC, ECC, and HCC for the U.S. were examined at the county-level. Further, the rates were mapped to illustrate the geographic burden of ICC, ECC, and HCC.

## Methods

### Incidence Data Source

County-level ICC, ECC, and HCC incidence rates were obtained from the North American Association of Central Cancer Registries (NAACCR) Cancer in North America (CiNA) data [9]. The CiNA dataset is a compilation of incidence data from the National Cancer Institute’s Surveillance, Epidemiology, and End Results and the Centers for Disease Control and Prevention’s National Program of Cancer Registries that provided active consent for the proposed study [9]. There were 33 registries that consented to participate and had complete data for 1998–2009. These 33 registries all met NAACCR gold or silver certification for complete, accurate, and timely data (Alabama, Alaska, Arizona, California, Colorado, Connecticut, Delaware, Florida, Georgia, Hawaii, Idaho, Illinois, Iowa, Kentucky, Louisiana, Maine, Massachusetts, Michigan, Minnesota, New Jersey, New Mexico, New York, North Dakota, Ohio, Oklahoma, Oregon, Pennsylvania, Rhode Island, South Carolina, Texas, Utah, Washington, and West Virginia). Approximately 75% of the U.S. population is covered by these 33 registries, comprising 1,970 counties.

### Modeled Incidence Rates

As previously described, a spatial-temporal model (PROC GLIMMIX, SAS 9.3, Cary, NC) was developed to estimate county-level incidence for the entire U.S. [10,11]. This model utilizes associations between observed incidence and mortality and the socioeconomic and demographic covariates to predict the numbers of cases estimated for each county [10,11]. Validation studies have shown that this model accurately predicts an estimated number of cases [12].

The model incorporated additional covariates related to these three cancer sites from multiple sources [10,11]. These included county-level mortality data provided from the National Center for Health Statistics [13] and the county-level rural-urban continuum code from the U.S. Department of Agriculture [14]. The model also incorporated U.S. Census Bureau covariates describing socio-economic demographic attributes at the county-level including age distribution, racial and ethnic population composition, income, poverty, education, and household characteristics [15]. Finally, the model accounted for county-level availability of physicians, cancer screening facilities, and health insurance from the Health Resources and Services Administration data [16] and tobacco use, obesity, physical activity, and cancer screening rates from the Behavioral Risk Factor Surveillance System [17].

Overall modeled U.S. incidence rates per 100,000 persons were estimated for ICC, ECC, and HCC. Rates were stratified by race and ethnicity (non-Hispanic Whites, non-Hispanic Blacks, Hispanics, non-Hispanic Asians/Pacific Islanders, and non-Hispanic American Indians/Alaska Natives), sex, age groups (0–35, 35–44, 45–54, 55–64, 65–74, 75–84, and 85+ years), and Census Tract Divisions (New England, Middle Atlantic, East North Central, West North Central, South Atlantic, East South Central, West South Central, and Mountain Pacific).

### Mapping

To illustrate the geographical distribution of incidence rates, modeled county estimates were mapped with ArcMap 10.1 (Environmental Systems Research Institute, Inc., Redland, CA). Site-specific quartiles were calculated for ICC and ECC (0.41–0.60, 0.61–0.70, 0.71–0.85, and >0.85 cases per 100,000 persons) and HCC (1.18–2.00, 2.01–3.00, 3.01–3.50, and >3.50 cases per 100,000 persons) utilizing the Jenks natural breaks optimization [18]. Modeled county estimates were mapped in quartiles using colors based on the ColorBrewer tool (ColorBrewer, Penn State, PA). Due to high predicted error, spatial smoothing was not applied to the final maps. Thus, the estimated rates in some counties with small case counts and populations could be unstable. We included state, division, and regional census partitions on the maps for ease of interpreting geographic variability.

### Incidence Trends

Joinpoint regression models (Joinpoint 3.5.0, IMS, Inc., Calverton, MD) were used to examine ICC, ECC, and HCC incidence trends in the 33 states with complete annual reporting data during 1998–2009.

## Results

**Table 1** presents demographic characteristics of ICC, ECC, and HCC cases in the U.S., during 2000–2009, based on the geospatial model. Overall HCC rates per 100,000 individuals were 4.23, nearly five times higher than rates for ICC (0.88) and nearly six times higher than ECC rates (0.72). Differences in rates across racial and ethnic groups were more pronounced for HCC than for ICC or ECC. For all three cancers, Asians/Pacific Islanders experienced the highest incidence rates (ICC: 1.28, ECC: 1.10, HCC: 11.81), followed by Hispanics (ICC: 1.16, ECC: 0.92, HCC: 8.36). American Indians/Alaska Natives had the third highest rates of ICC and ECC (0.98 and 0.72, respectively), followed by whites (0.84 and 0.69, respectively) and then blacks (0.83 and 0.64, respectively). Blacks had the third highest rates of HCC (6.23), followed by American Indians/Alaska Natives (5.92) and finally, whites (3.22). For all three cancer sites men had higher rates than women, however the male to female ratio was more than three-fold for HCC (3.8 to 1) but less than two-fold for ICC (1.4 to 1) and ECC (1.5 to 1). For both ICC and ECC, peak rates occurred among persons 75 years of age and older. ECC incidence rates exceeded those for ICC in one age group only, persons 85 years of age and older (5.84 versus 5.43, respectively). Peak rates for HCC were seen in the 65–74 (16.62) and 75–84 (17.39) year age groups. Modeled ICC rates were highest in New England and ECC rates were highest in the Middle Atlantic division, while HCC incidence rates were highest in the Pacific division.

**Table 1:** Modeled age-adjusted hepatocellular carcinoma, intrahepatic cholangiocarcinoma, and extrahepatic cholangiocarcinoma incidence rates per 100,000; 2000–2009 by demographic group.

	Intrahepatic cholangiocarcinoma		Extrahepatic cholangiocarcinoma		Hepatocellularcarcinoma	
	Count	Modeled Rate	Count	Modeled Rate	Count	Modeled Rate
**Race/Ethnicity** ^**a**^						
White	16,069	0.84	13,589	0.69	63,712	3.22
Black	1,850	0.83	1,389	0.64	15,070	6.23
Hispanic	2,327	1.16	1,757	0.92	18,172	8.36
Asians/Pacific Islanders	1,200	1.28	1,014	1.10	11,732	11.81
American Indians/Alaska Natives	132	0.98	95	0.72	954	5.92
**Sex**						
Male	11,195	1.04	9,471	0.89	83,541	7.05
Female	10,383	0.76	8,373	0.58	26,099	1.85
**Age (Years)**						
0–35	243	0.03	96	0.01	1,360	0.12
35–44	781	0.18	440	0.09	3,544	1.18
45–54	2,676	0.80	1,487	0.43	23,741	6.24
55–64	4,668	2.04	3,143	1.37	30,803	12.39
65–74	5,715	3.63	4,751	3.05	25,757	16.62
75–84	5,485	5.14	5,546	5.00	19,912	17.39
85+	2,010	5.43	2,381	5.84	4,523	11.85
**Census Division**						
New England	1,372	0.99	1,099	0.79	5,887	4.15
Middle Atlantic	4,205	0.94	3,681	0.82	21,256	4.74
East North Central	3,151	0.90	2,610	0.74	12,366	3.37
West North Central	428	0.94	365	0.68	1,199	3.03
South Atlantic	3,380	0.80	2,733	0.63	15,309	3.63
East South Central	1,192	0.81	867	0.58	5,129	3.30
West South Central	2,749	0.94	1,951	0.68	15,800	5.25
Mountain	1,105	0.74	1,032	0.68	5,970	3.77
Pacific	3,996	0.90	3,506	0.79	26,724	5.94
**Overall**	21,578	0.88	17,844	0.72	109,640	4.23

^a^All races are non-Hispanic.

**Table 2** presents joinpoint regression incidence trends during 1998–2009 for the 33 registries that contributed complete data for these years. For all three cancer sites the best fitting model included one joinpoint and two trends. For ICC, incidence rates declined during 1998–2003 (annual percent change [APC] = -8.1% per year, 95% confidence interval [CI]: -13.8, -2.1) and increased from 2003–2009 (APC = 5.9% per year, 95% CI: 1.2, 10.8). In contrast, ECC incidence rates increased during 1998–2003 (APC = 9.1% per year, 95% CI: 4.5, 14.0) and stabilized from 2003–2009 (APC = 1.1% per year, 95% CI: -1.7, 3.9). The average annual percent change (AAPC) in ICC incidence increased during the most recent five years: 2005–2009, 5.9% (95% CI: 1.2, 10.8). For ECC the AAPC increased during the most recent ten years: 2000–2009, 3.7% (95% CI: 1.7, 5.8). Rising incidence trends for HCC decelerated during 2007–2009 (APC = 3.3% per year, 95% CI: 1.4, 5.3) compared to 1998–2007 (APC = 5.5% per year, 95% CI: 5.3, 5.7).

**Table 2:** Hepatocellular carcinoma, intrahepatic cholangiocarcinoma, and extrahepatic cholangiocarcinoma incidence trends, 1998–2009.

							2005–2009		2000–2009	
	Trend 1	APC^a^	95% CI	Trend 2	APC^a^	95% CI	AAPC^b^	95% CI	AAPC^b^	95% CI
Intrahepatic cholangiocarcinoma	1998–2003	-8.1*	-13.8, -2.1	2003–2009	5.9*	1.2, 10.8	5.9*	1.2, 10.8	1.0	-2.0, 4.1
Extrahepatic cholangiocarcinoma	1998–2003	9.1*	4.5, 14.0	2003–2009	1.1	-1.7, 3.9	1.1	-1.7, 3.9	3.7*	1.7, 5.8
Hepatocellular carcinoma	1998–2007	5.5*	5.3, 5.7	2007–2009	3.3*	1.4, 5.3	4.4*	3.6, 5.2	5.0*	4.6, 5.4

^a^APC = annual percent change.

^b^AAPC = average annual percent change.

*Significant at p<0.05.

**Fig 1:**
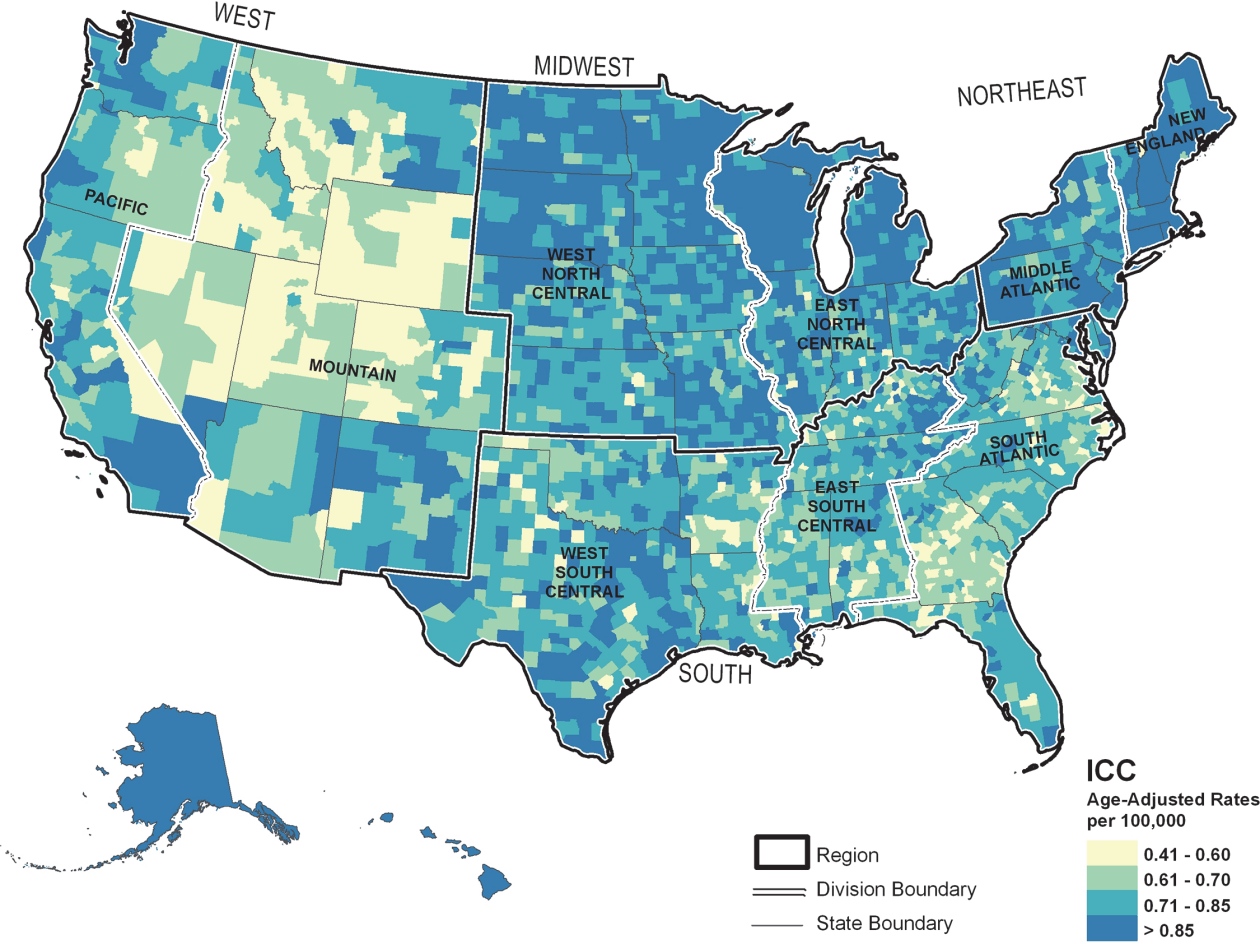
Modeled United States county-level intrahepatic cholangiocarcinoma incidence rates, 2000–2009.

In **Fig 1**, mapped ICC incidence rates are shown. The lowest rates of ICC, approximately 0.41 to 0.60 per 100,000 individuals, were primarily seen in rural counties of the northern and central Rocky Mountain States and in the South. The highest ICC incidence rates, >0.85 per 100,000 individuals, were seen in the Northeast and upper Midwest and in the southwest from Texas to California and coastal counties of the Pacific, including Hawaii and Alaska.

**Fig 2:**
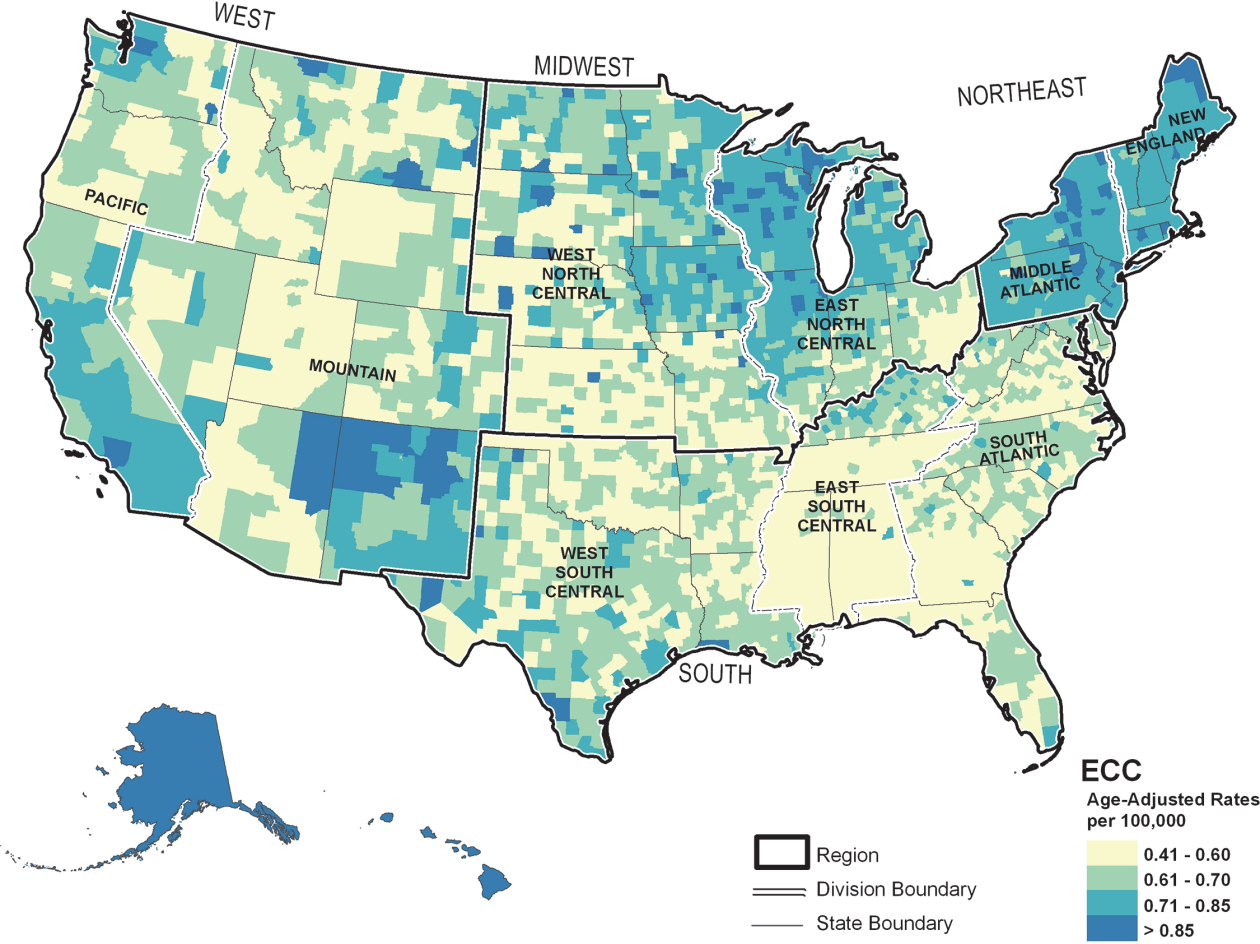
Modeled United States county-level extrahepatic cholangiocarcinoma incidence rates, 2000–2009.

Mapped ECC incidence rates resembled those of ICC, but the pattern was more diffuse (**Fig 2**). The lowest rates of ECC, approximately 0.41 to 0.60 per 100,000 individuals were seen in rural counties of the northern and central Rocky Mountain States and the South. The highest ECC incidence rates, >0.70 per 100,000 individuals, were seen in the Northeast and upper Midwest and in the southwest from Texas to California and the Pacific region, including Hawaii and Alaska.

**Fig 3:**
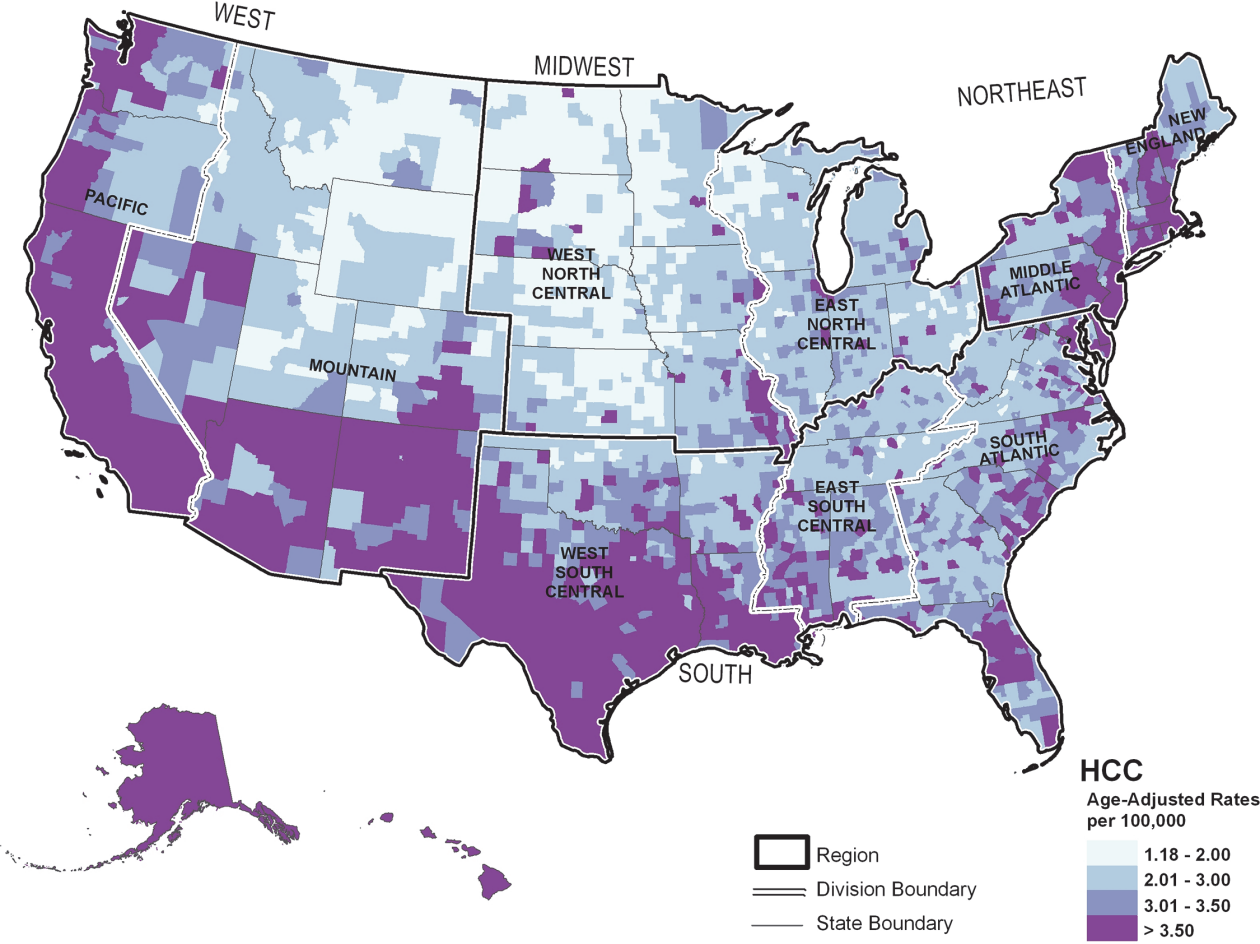
Modeled United States county-level hepatocellular carcinoma incidence rates, 2000–2009.

**Fig 3** shows mapped HCC incidence rates. The lowest rates of HCC, approximately 1.18 to 2.00 per 100,000 individuals, occurred primarily in inland rural counties. This pattern was most pronounced in the northern states of the West and Midwest. The highest HCC incidence rates, >3.50 per 100,000 individuals, were seen in coastal and southern states, often centered on urban counties.

Common features of the distribution of all three cancer sites included the presence of intermediate rates in counties adjacent to both low and high incident areas and high incidence rates in Hawaii and Alaska.

## Discussion

In the current study, overall rates, trends, racial distribution, male:female ratio, and peak ages were more similar between ICC and ECC than HCC. While geographical variation is noted between cancer sites, the geographical patterns between ICC and ECC were more closely related. High incidence for all three cancer sites was found in the Pacific region, particularly Hawaii and Alaska, suggesting a need for cancer control efforts in this area. However, the geographic variability between cancer sites suggests additional need for ICC and ECC control efforts focused in the Northeast and upper Midwest, while HCC control efforts should be additionally focused in the South.

Geographical variation of disease is often explained by differences in demographics and environmental risk factors, which suggest that risk factors for HCC may be more dissimilar than those of ICC and ECC. For instance Hispanics, Asians/Pacific Islanders, and Blacks were over-represented among HCC cases compared to their proportions in the general population. Race and ethnicity appeared to be less of a factor in the geographical patterns of ICC and ECC. In particular, approximately 75% of all ICC and ECC cases occurred among non-Hispanic whites (compared to 58% of HCC cases). Other established risk factors for ICC and ECC, such as Caroli’s disease, primary sclerosing cholangitis, and inflammatory bowel disease, could partially explain geographical similarities between the two tumor types [6]. Another possibility is that potential misclassification of ICC and ECC have made determining distinct geographical patterns for each site difficult [19].

In this study, the highest rates for ICC, ECC, and HCC were seen in Asians/Pacific Islanders. However, it is unclear why Asians/Pacific Islanders have the highest rates of all three types of cancers. This could potentially be due to a common set of risk factors specific to Asians/Pacific Islanders that contribute to each of these cancers. For instance, rates of HCC have historically been highest among Asians/Pacific Islanders due to higher rates of chronic hepatitis B virus (HBV) infection among older individuals born outside of the U.S. [20]. A recent meta-analysis has also shown that HBV is associated with an increased risk of ICC and, to a lesser extent, ECC [21]. Thus, HBV infection could potentially account for the high incidence rates of HCC, ICC, and ECC seen among Asians/Pacific Islanders in the present study. Additionally, approximately 46% of the Asians/Pacific Islanders in the U.S. live in the Pacific region [22], which is likely to partially account for the high rates of these cancers seen in this region.

Rates among racial/ethnic groups other than Asians/Pacific Islanders vary by cancer site. For HCC, whites have notably lower rates than those of other racial/ethnic groups. For ICC and ECC, the rates among whites, blacks, and American Indians/Alaska Natives did not vary greatly. The greater variability in racial and ethnic distribution among HCC than ICC and ECC cases is perhaps due to differences in exposure or susceptibility to HCC-unique risk factors. In addition, societal factors of cancer prevention, detection, or treatment are hypothesized to contribute to overall higher incidence of all cancer among blacks [23]. Thus, efforts should be focused on identifying and targeting race-specific risk factors for HCC and the more homogeneous risk factors for ICC and ECC.

Higher rates of all three cancer types were seen among individuals over 75 years of age and males. While sex-differences of these cancers are not completely understood, it is hypothesized that the higher prevalence of risk factors (e.g., HBV/HCV, alcohol consumption, and potentially androgenic hormones) among males could partially account for these differences [24]. The high incidence in older age groups is thought to be partially due to HCV infection, a risk factor for HCC and possibly ICC, as most individuals become infected with HCV as adults [24]. HCV infection is most prevalent among individuals born between 1945 and 1965 (i.e., baby-boomers). While these individuals do not have the highest rates of liver cancer, possibly due to lack of sufficient latency period for cancer progression, they do have the most rapidly increasing incidence rates [25].

The direction of incidence trends differed for the three cancer types. HCC incidence decreased during 2007–2009, compared to 1998–2007. This is consistent with data in SEER registries, which found a deceleration in increasing rates among baby-boomers and decreasing rates among younger adults [26]. Incidence trends for ECC increased during 2000–2009 while ICC incidence trends initially decreased then increased from 2007–2009, yielding a stable trend during 2000–2009. In one study [6], HCV infection, chronic nonalcoholic liver disease, obesity, and smoking were associated with ICC but not ECC, suggesting that risk factors could explain divergent incidence trends for these highly fatal tumors. Another possible explanation for divergent trends is that recent ICD-O coding changes may skew cholangiocarcinoma classification toward C22 topography, contributing to the recent rise in ICC and deceleration in ECC incidence trends [19].

A limitation of this study is the potential for misclassification of race or ethnicity. Hispanics were analyzed separately from other racial/ethnic groups; however, the ethnicity of “Hispanic” is very broad and encompasses Mexicans, Puerto Ricans, and Cubans, as well as other individuals that identify with “another Hispanic, Latino, or Spanish origin” [27]. Thus, these individuals could have widely varying genetic, environmental, cultural, and socioeconomic risk factors [23]. Additionally, known risk factors for HCC (e.g., HBV/HCV prevalence and diabetes) were not accounted for in the spatial-temporal modeling.

A strength of this study is the utilization of the NAACCR CiNA database. While seventeen states and the District of Columbia either did not agree to utilization of data or did not have complete data for the study period, this data provides the most comprehensive population coverage of the U.S. to date. For areas that did not provide data, a validated spatial-temporal model was utilized to estimate rates [12]. Thus, geographic patterns for the entire U.S. were able to be analyzed.

While HCC and ICC may have some common etiologies, such as obesity, chronic non-alcoholic liver disease, and tobacco [6], geographic areas with high ICC incidence do not completely correspond with areas of high HCC incidence. These unique geographic distributions could suggest differences in etiology. In the current study, demographic patterns and geographical variation were more closely related for ICC and ECC than HCC. This study suggests a need for cancer control efforts focused in the Pacific region, particularly Hawaii and Alaska, for all three cancer sites. While additional control efforts for ICC and ECC should be focused in the Northeast and upper Midwest, HCC control efforts should be additionally focused in the South. Findings of geographic variation in incidence rates for these cancer sites may help target etiologic and cancer control efforts.
